# Comparison of sodium and potassium measurements in the emergency department by point-of-care vs. central laboratory testing: A retrospective analysis

**DOI:** 10.1007/s00508-026-02741-9

**Published:** 2026-04-13

**Authors:** Svenja Ravioli, Annemarie Edenhofner, Christoph Schwarz, Susanne Oswald, Gregor Lindner

**Affiliations:** 1 Department of Emergency Medicine, Kepler Universitätsklinikum GmbH, Johannes Kepler University, Krankenhausstrasse 9, 4020 Linz, Austria; 2 Department of Internal Medicine, Pyhrn-Eisenwurzen Klinikum Steyr, Steyr, Austria; 3 Department of Emergency Medicine, Inselspital, University of Bern, Bern, Switzerland

**Keywords:** Electrolytes, Emergency, POCT, Potassium, Sodium

## Abstract

**Background:**

Point-of-care testing (POCT) using blood gas analyzers provides rapid results and can reduce diagnostic delays. Discrepancies between POCT and central laboratory (CL) measurements have been observed, particularly in patients with sodium disorders. This study evaluated the agreement between POCT and CL measurements for sodium and potassium in patients with hyponatremia, normonatremia and hypernatremia.

**Methods:**

In this retrospective single-center analysis, all paired measurements of sodium and potassium performed by POCT (Radiometer ABL 90 Flex Plus) and the CL (Roche Cobas Pro ISE) within ± 2 h between January and December 2024 were included. The correlation, Bland-Altman analysis, paired tests and intraclass correlation coefficients (ICC) were used to assess agreement. Regression analysis evaluated proportional bias and ANOVA tested differences among dysnatremic and normonatremic groups.

**Results:**

A total of 6404 paired sodium and 5622 paired potassium measurements were analyzed. Sodium was 140.3 mmol/L (SD 4.5) (POCT) and 137.8 mmol/L (SD 6.6) (CL), showing a mean difference of 2.4 mmol/L (SD 1.5). The correlation was significant (r = 0.583, *p* < 0.001) but ICC indicated poor agreement (0.473). The ANOVA demonstrated statistically significant differences between sodium groups (*p* = 0.007, effect size 0.002), indicating more pronounced deviations in hypernatremic patients. For potassium, the mean difference was 0.12 mmol/L (interquartile range, IQR 0.05–0.22 mmol/L) with good agreement (ICC = 0.85) but a significant proportional bias (*p* < 0.001). Clinically relevant deviations (> 4 mmol/L sodium or > 0.5 mmol/L potassium) occurred in 8.5% and 4.5% of cases, respectively.

**Conclusion:**

While potassium showed a very strong correlation and good agreement, sodium demonstrated only moderate correlation and poor agreement according to ICC criteria. Systematic and clinically relevant deviations were frequent, particularly in dysnatremic and dyskalemic patients.

## Background

Overcrowding is an increasingly worrisome challenge in emergency medicine, characterized by a mismatch between demand and supply. It is expressed by long waiting times and overfilled waiting zones in emergency departments potentially compromising patient safety [1]. Point-of-care testing (POCT) is one suggested way to reduce waiting times for diagnostics and thus patient time in the department in order to reduce the burden of overcrowding [1].

Blood gas analyzers are often used in the emergency medicine setting as they enable rapid determination of electrolytes, hemoglobin, glucose, lactate and acid-base parameters. Studies investigating the reliability and reproducibility of results gained by whole blood analysis via blood gas analyzers compared to serum determination in central laboratories resulted in significant differences, which were classified as being clinically irrelevant [2–4]. Furthermore, it has been empirically observed that in patients with relevant hyponatremia or hypernatremia results of POCT for serum sodium tend to relevantly deviate from central laboratory results. This is of high importance since both hyponatremia and hypernatremia are associated with high morbidity and mortality and are commonly observed in hospitalized patients, especially those admitted to the emergency department [5–8]. In addition, treatment recommendations rely on intervals of serum sodium as narrow as a few mmol/L [9]. So far, only one single study evaluated the agreement for serum sodium results between whole blood analysis as in blood gas analyzers and serum measurements as applied in central laboratories [10]. This study found high deviations, which cannot be considered clinically irrelevant [10]. Given that only one single center study exists on this important issue, it is obvious that there is a fundamental lack of data even though these methods are regularly used in the daily routine.

Therefore, in the present study we aimed to evaluate the agreement between measurements of sodium and potassium in patients with hyponatremia, normonatremia and hypernatremia/hypokalemia, normokalemia and hyperkalemia between POCT by blood gas analyzer and core laboratory measurement.

## Patients, material and methods

### Study design and parameters

In this retrospective analysis, all measurements of sodium and potassium from venous whole blood by the Radiometer ABL 90 Flex Plus (Radiometer Medical ApS, Copenhagen, Denmark) performed in the Department of Emergency Medicine with corresponding measurement of serum sodium and potassium in the central laboratory within a time window of ± 2 h between 1 January 2024 and 31 December 2024 were obtained. Usually, samples were taken from the same blood drawing minimizing time differences. Samples for the central laboratory were taken exclusively from venous blood. Central laboratory analysis was performed on plasma samples by use of the Roche Cobas Pro ISE (ion selective electrode; Roche, Basel, Switzerland). Almost exclusively, these were samples from one blood drawing.

In addition, data concerning patient sex and age were gathered.

### Statistical analysis

All analyses were performed using JASP 0.19.3 (JASP, Amsterdam, Netherlands). Data are presented as mean and standard deviation or median and interquartile range (IQR) as appropriate.

In order to test for correlation, Pearsonʼs correlation coefficient for normally distributed data or Spearman’s rank correlation for non-parametric data was applied.

The strength of Pearson’s correlation was interpreted as follows: 0.00–0.19 very weak, 0.20–0.39 weak, 0.40–0.59 moderate, 0.60–0.79 strong, and ≥ 0.80 very strong. Bland-Altman plots were used to test for agreement between the two measurements.

Paired t‑tests and Wilcoxon signed-rank tests were used to test for statistical significance. Moreover, we used intraclass correlation coefficients (ICC) to quantify degrees of absolute agreement. The ICC values were interpreted according to Koo and Li: < 0.5 poor, 0.5–0.75 moderate, 0.75–0.9 good, and > 0.9 excellent agreement [11]. In order to assess for proportional bias, i.e. varying differences between POCT and central laboratory with changing absolute sodium values, we performed a regression analysis for the difference and the mean of the two measurements as well as Bland-Altman plots. The ANOVA was used to calculate whether a difference could be observed between the respective electrolyte measurements for the respective groups of hyponatremia, normonatremia and hypernatremia/hypokalemia, normokalemia and hyperkalemia.

### Ethical considerations

The study was approved by the local institutional review board, the Ethics Committee of the Johannes Kepler University. The need for individual patient consent was waived.

## Results

During the study period a total of 34,215 cases presented to the Department of Emergency Medicine of the Kepler University Hospital. Mean time difference between venous sampling for POCT and central laboratory samples was < 1 min with a standard deviation (SD) of 0.7. A time difference > 10 min between taking of the two samples occurred only in one case.

### Sodium measurements

A total of 6404 patients had measurements of sodium by POCT as well as by the central laboratory within 2 h. The mean age of patients was 63 years (SD 21) and 3314 (52%) were women.

**Fig. 1:**
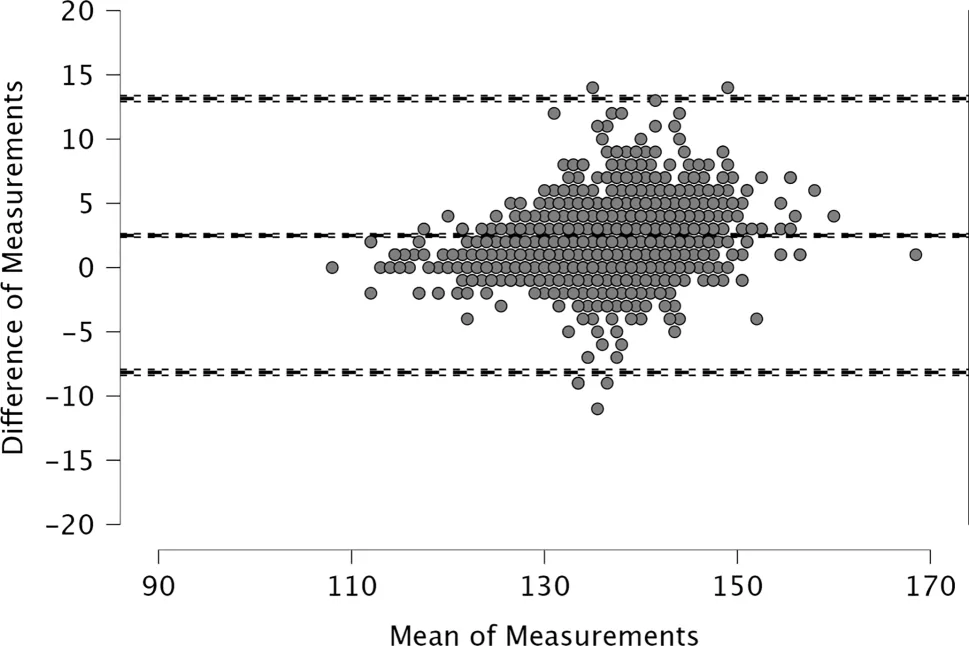
Bland-Altman plot for the correlation of sodium measurement between POCT and central laboratory. values in mmol/L. Horizontal lines are mean and 95%CI

The mean sodium measured in the central laboratory was 137.8 mmol/L (SD 6.6) while it was 140.3 mmol/L (SD 4.5) in the POCT. The mean difference between sodium determination by POCT vs. central laboratory was 2.4 mmol/L (SD 1.5). There was a statistically significant moderately positive correlation (Pearson’s r 0.583, *p* < 0.001, 95% CI 0.566–0.599). The intraclass correlation coefficient (ICC) between POCT and central laboratory measurement was 0.473 (95% CI 0.454–0.492), indicating poor correlation according to Koo and Li [11]. Figure 1 provides a Bland-Altman plot for the correlation of POCT and central laboratory sodium measurements. Regression analysis to assess for proportional bias showed a positive correlation coefficient for the mean of 0.217 (*p* < 0.001, 95% CI 0.081–0.101) indicating systematic bias in the correlation between the two modes of measurement.

In the central laboratory 5379 samples were normonatremic (135–145 mmol/L), 946 (15%, 95% CI 13.9–15.7%) were hyponatremic and 79 (1.3%, 95% CI 0.99–1.52%) were hypernatremic. The median absolute difference between POCT and central laboratory measurements was 2 mmol/L (IQR 1–3 mmol/L) in hyponatremic patients and 2 mmol/L (IQR 1–4 mmol/L) in hypernatremic patients; however, when analyzing mean absolute differences, hypernatremic patients showed the highest deviations (mean 2.72 mmol/L, SD 1.9), followed by normonatremic (2.47 mmol/L, SD 1.5) and hyponatremic patients (2.32 mmol/L, SD 1.9). The ANOVA demonstrated a statistically significant difference between the groups (*p* = 0.007, effect size 0.002), indicating that deviations were overall most pronounced in hypernatremic patients. Table 1 provides details of the ANOVA.

**Table 1:** ANOVA results for differences in sodium between POCT and central laboratory testing

Descriptives—Absolute difference					
Sodium group	*N*	Mean	SD	SE	Coefficient of variation
Hypernatremia	79	2.722	1.901	0.214	0.699
Hyponatremia	946	2.315	1.920	0.062	0.829
Normonatremia	5379	2.470	1.523	0.021	0.617

In 545 cases (8.5%), the difference between POCT and central laboratory testing was > 4 mmol/L and in 31 (0.5%) cases it was > 8 mmol/L.

### Potassium measurements

During the study period a total of 5622 measurements of potassium by POCT with corresponding measurements in the central laboratory within a 2 h time window were available for analysis. The mean age of patients was 62 years (SD 21) and 2891 (51%) were women.

**Fig. 2:**
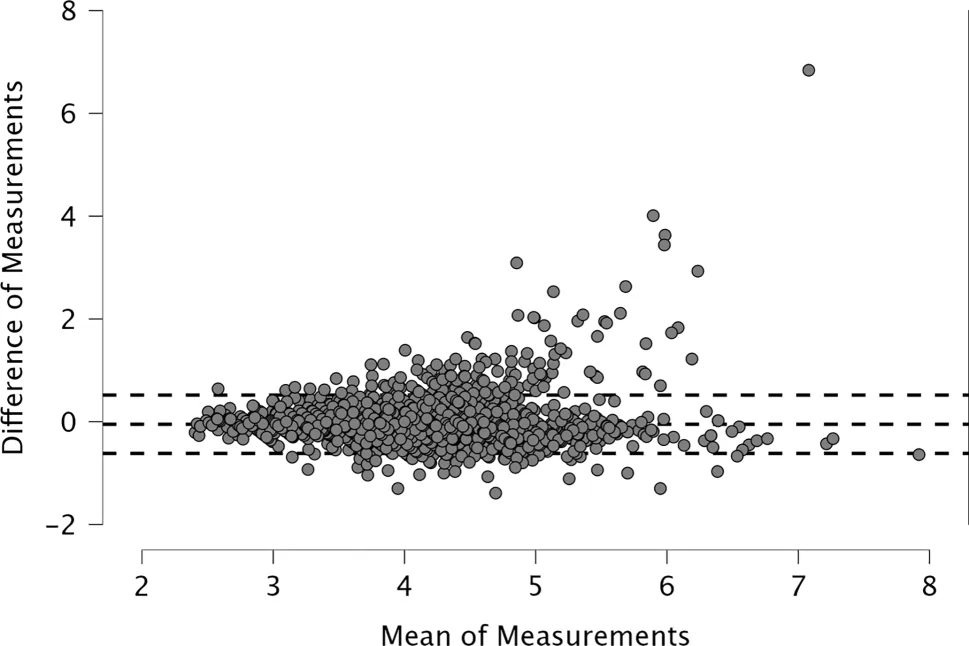
Bland-Altman plot for the correlation of potassium measurement between POCT and central laboratory. Values in mmol/L

The mean potassium in the central laboratory was 4.046 mmol/L (SD 0.531) while it was 3.997 mmol/L (SD 0.541) as measured by POCT. The median difference between potassium measurement by POCT and central laboratory was 0.12 mmol/L (IQR 0.05–0.22 mmol/L). There was a statistically significant very strong positive correlation between the two types of potassium measurements with a Pearson’s r of 0.854, *p* < 0.001, 95% CI 0.847–0.861. The ICC between POCT and central laboratory measurement was 0.85 (95% CI 0.843–0.857), meaning good correlation. Figure 2 provides a Bland-Altman plot for potassium measurement by POCT and central laboratory.

Regression analysis to assess for proportional bias showed a positive correlation coefficient for the mean of 0.151 (*p* < 0.001, 95% CI 0.14–0.162) indicating systematic bias in the correlation between the two modes of measurement.

Of the samples 4710 showed normokalemia defined as a potassium between 3.5 and 5.0 mmol/L in the central laboratory. 642 samples (11% of total, 95% CI: 10.6–12.3%) were hypokalemic (i.e. [K^+^]< 3.5 mmol/L) in the central laboratory. Of the samples 270 (4.8%, 95% CI: 4.3–5.4%) were hyperkalemic in the central laboratory (i.e. [K^+^]> 5.0 mmol/L). ANOVA analysis showed a highly significant difference between results of POCT versus central laboratory potassium measurements, (*p* < 0.001, effect size 0.023) with deviations again being smallest in the hypokalemic group and largest in the hyperkalemic group. Table 2 provides details of the ANOVA.

**Table 2:** ANOVA results for differences in potassium between POCT and central laboratory testing

Descriptives—Difference					
Potassium group	*N*	Mean	SD	SE	Coefficient of variation
Hyperkalemia	270	0.325	0.279	0.017	0.860
Hypokalemia	642	0.133	0.188	0.007	1.415
Normokalemia	4710	0.171	0.236	0.003	1.381

In 254 cases (4.5%) the difference between POCT and central laboratory was > 0.5 mmol/L and in 56 (1%) cases it was > 1 mmol/L.

## Discussion

The present study aimed to assess differences in measurements of sodium and potassium either from whole blood by POCT or from serum in the central laboratory. The study explicitly not only examined absolute differences but clinically relevant scenarios, such as differences in patient groups with electrolyte disorders and frequencies of clinically relevant deviations.

Overall, the analysis of > 5500 samples from patients admitted to the emergency department showed only small, clinically irrelevant deviations between POCT measurement and central laboratory determination of sodium and potassium; however, more importantly, the present study brings two important new facts to light: Firstly, systematic bias was found for sodium and potassium measurements with greater differences between POCT and central laboratory measurements with increasing absolute levels of sodium and potassium. For example, clinically relevant deviations of 0.5 mmol/L in hyperkalemia patients are present in > 15% of cases, while in 4.5% overall. Secondly, even more importantly, it was found that a clinically relevant difference between the two modes of measurement was identified in a significant number of patients: for potassium, the difference exceeded 0.5 mmol/L in as many as 4.5% of patients while in 8.5% of sodium measurements the difference exceeded 4 mmol/L. This poses significant challenges for the management of both disorders of serum potassium as well as serum sodium.

In the acute management of hyponatremia with moderate to severe symptoms, the administration of hypertonic saline is recommended [9, 12]. A control of sodium is recommended after administration of hypertonic saline and a repeat dose is indicated if symptoms persist and sodium did not rise by > 5 mmol/L [12]. This highlights the importance of valid results for control values, especially in the acute setting, which is questioned by the present results. Similar considerations are true for the controls of potassium during the management of potassium disorders, acute hyperkalemia in particular [13, 14]. On the basis of the findings of the present study, it appears justified to recommend management of electrolyte disorders using the same mode of electrolyte determination during active correction phases, e.g. if baseline sodium measurement was performed in the central laboratory, control values should be obtained by the same method.

Our present findings are supported by a recent study, which showed overall significant correlation and high reliability of POCT and central laboratory measurements; however, the study collective was small with only 120 patients [15]. Other, previously performed studies found significant differences in electrolyte measurements between POCT and laboratory testing [16]. Of note, patients with dysnatremia and dyskalemia were not specifically analyzed in previous studies.

Our study has several limitations: Firstly, it is a retrospective analysis and data were not specifically obtained for scientific purposes. Secondly, for the present analysis we do not have data to analyze potential causes of significant differences between the modes of measurement in the patient groups with clinically relevant sodium and potassium differences. Thirdly, we cannot exclude technical issues during short periods of POCT use, although it should be clearly outlined that service intervals are narrow and analyzer checks are performed daily by the emergency department staff. In addition, it has to be kept in mind that the methods are limited in comparability: while POCT uses whole blood, central laboratory uses plasma for electrolyte determination. Therefore, factors such as hematocrit variation, protein concentration, lipemia or hemolysis are not available in the dataset but might contribute to systematic bias. Moreover, the population of hypernatremic patients was small and thus the results must be interpreted with caution.

In conclusion, the present study comparing measurements of sodium and potassium by POCT versus central laboratory testing in a large population of patients admitted to the emergency department showed a very strong correlation between POCT and central laboratory for potassium measurement, while only a moderate correlation for sodium, indicating lower agreement between methods for sodium; however, a significant bias was found both for sodium as well as potassium measurements between POCT and laboratory determination, especially in dysnatremic and dyskalemic patients. Clinically relevant differences in sodium and potassium were identified in a significant proportion of patients. This should be cause for concern for emergency physicians in the care of patients with disorders of sodium and potassium.

## Data Availability

The data used in this study can be requested from the authors on justified cause.

## Abbreviations

CL: Central laboratory
ICC: Intraclass correlation coefficient
POCT: Point of care testing
SD: Standard deviation
